# The attention-emotion interaction in healthy female participants on oral contraceptives during 1-week escitalopram intake

**DOI:** 10.3389/fnins.2022.809269

**Published:** 2022-09-09

**Authors:** Nathalie Beinhölzl, Eóin N. Molloy, Rachel G. Zsido, Thalia Richter, Fabian A. Piecha, Gergana Zheleva, Ulrike Scharrer, Ralf Regenthal, Arno Villringer, Hadas Okon-Singer, Julia Sacher

**Affiliations:** ^1^Emotion and Neuroimaging Lab, Max Planck Institute for Human Cognitive and Brain Sciences, Leipzig, Germany; ^2^Department of Neurology, Max Planck Institute for Human Cognitive and Brain Sciences, Leipzig, Germany; ^3^University Clinic for Radiology and Nuclear Medicine, Otto Von Guericke University Magdeburg, Magdeburg, Germany; ^4^German Center for Neurodegenerative Diseases, Magdeburg, Germany; ^5^Max Planck School of Cognition, Leipzig, Germany; ^6^Department of Psychology, School of Psychological Sciences, University of Haifa, Haifa, Israel; ^7^The Integrated Brain and Behavior Research Center (IBBRC), University of Haifa, Haifa, Israel; ^8^Division of Clinical Pharmacology, Rudolf Boehm Institute of Pharmacology and Toxicology, University Leipzig, Leipzig, Germany; ^9^Clinic for Cognitive Neurology, University Hospital Leipzig, Leipzig, Germany; ^10^Berlin School of Mind and Brain, MindBrainBody Institute, Charité—Berlin University of Medicine and Humboldt University Berlin, Berlin, Germany; ^11^Department of Psychiatry, Psychosomatic Medicine and Psychotherapy, Helios Park Hospital Leipzig, Leipzig, Germany

**Keywords:** selective serotonin reuptake inhibitors, attention-emotion interaction, oral hormonal contraceptives, female mental health, emotional flanker task

## Abstract

Previous findings in healthy humans suggest that selective serotonin reuptake inhibitors (SSRIs) modulate emotional processing *via* earlier changes in attention. However, many previous studies have provided inconsistent findings. One possible reason for such inconsistencies is that these studies did not control for the influence of either sex or sex hormone fluctuations. To address this inconsistency, we administered 20 mg escitalopram or placebo for seven consecutive days in a randomized, double-blind, placebo-controlled design to sixty healthy female participants with a minimum of 3 months oral contraceptive (OC) intake. Participants performed a modified version of an emotional flanker task before drug administration, after a single dose, after 1 week of SSRI intake, and after a 1-month wash-out period. Supported by Bayesian analyses, our results do not suggest a modulatory effect of escitalopram on behavioral measures of early attentional-emotional interaction in female individuals with regular OC use. While the specific conditions of our task may be a contributing factor, it is also possible that a practice effect in a healthy sample may mask the effects of escitalopram on the attentional-emotional interplay. Consequently, 1 week of escitalopram administration may not modulate attention toward negative emotional distractors outside the focus of attention in healthy female participants taking OCs. While further research in naturally cycling females and patient samples is needed, our results represent a valuable contribution toward the preclinical investigation of antidepressant treatment.

## Introduction

Selective serotonin reuptake inhibitors (SSRIs) are the first line pharmacological treatment for major depressive and anxiety disorders (Cleare et al., 2015; Hieronymus et al., 2018). While the key molecular mechanisms of SSRI action are relatively well understood, with occupancy of the serotonin transporter (Artigas et al., 2002), resulting in an upregulation of serotonin neurotransmission, the specific mechanism by which SSRIs improve mood still remains unclear. Moreover, though upregulation of serotonin neurotransmission occurs within a relatively short timeframe, clinically relevant changes in mood often take up to several weeks to manifest (Frazer and Benmansour, 2002). Recent findings in healthy participants (Harmer et al., 2004, 2011) and depressed patients (Harmer et al., 2009b; Roiser et al., 2012) have shed some light on this apparent temporal inconsistency by suggesting that SSRIs may modulate implicit emotional and social processing via earlier changes in selective attention (Harmer et al., 2009a). Critically, these attentional changes are hypothesized to *precede* improvements in mood via modulated processing of emotional stimuli and other downstream neuroadaptive effects (Harmer et al., 2017). In particular, evidence suggests that antidepressant action may be attributed to early changes in biased orienting of attention to aversive stimuli, a process that rebalance negative affective bias by increasing the relative recognition of positive over negative stimuli. On this account, the proposed delay is largely mediated by translation of changes in emotional bias to improved mood. While this model is promising, findings are inconsistent and remains to be fully evaluated.

Several lines of evidence have provided support for this attentional-emotional interplay. For example, evidence from neuroimaging studies indicates an SSRI-induced alteration in limbic blood oxygenation level dependent (BOLD) activation in response to emotional stimuli in healthy participants, showing a decrease of activation toward aversive stimuli (Harmer et al., 2006; Murphy et al., 2009a; Sladky et al., 2015), and an increase in response to positive stimuli (Norbury et al., 2009; Outhred et al., 2014). More recent research has presented evidence that a single 20 mg dose of escitalopram can blunt neural responses to averse stimuli in health (Lewis et al., 2021). Moreover, attentional networks are modulated by SSRI-administration, specifically, by increasing the engagement of the medial and dorsolateral prefrontal cortices (Fales et al., 2009; Ma, 2015), regions known for facilitating selective attention (Ochsner and Gross, 2005). Additionally, attentional bias training shows changes in emotional processing, measured by reduced amygdala reactivity to aversive information and changes in amygdala-prefrontal cortex connectivity (Cohen et al., 2016). Behavioral findings, however, remain comparatively inconsistent. While some studies have shown altered processing of emotional stimuli following subacute SSRI intake (Harmer et al., 2003, 2004), others have reported contrary outcomes, and did not find an effect of SSRI-administration on processing of affective stimuli (Skandali et al., 2018) nor on inhibitory performance and response re-engagement (Drueke et al., 2010) in healthy humans. Browning et al. (2007b), for example, reported that acute administration of 20 mg citalopram may draw participants attention toward positive words, but also enhances recognition of anxiety-related stimuli in healthy volunteers. Additionally, further findings also show that 7 days of 20 mg citalopram induces a decrease in memory for negative information in health (Browning et al., 2011). Another study, however, support the reversing effect of SSRIs on cognitive biases, showing that 1 week of 20 mg citalopram reduces attentional orienting to threatening stimuli, in contrast to reboxetine, a selective noradrenergic reuptake inhibitor (Murphy et al., 2009b). Rose et al. (2006) reported that 7 days administration of 10 mg escitalopram did not have a significant influence on cognitive flexibility, auditory selective attention, verbal learning and recall. Consequently, findings in healthy subjects have been inconclusive, thus presenting a significant barrier toward translating these results to patient samples.

These mixed findings may be a result of varying study designs or other overlooked contributing factors, such as small sample sizes, statistical power, selection bias, and the various distinct methodological approaches between studies. One largely overlooked variable, however, for example is sex. In a systematic review of 51 placebo-controlled trials in healthy participants (Knorr et al., 2019), many studies exhibited unequal sex distribution (62% male and 38% female) and the reported findings were thus largely contradictory. This disparity in sex among participants may be a significant contributing factor, given that sex, sex hormones, and the menstrual cycle are known to modulate both serotonergic signaling (Barth et al., 2015) and SSRI responsivity (LeGates et al., 2019). Moreover, menstrual cycle phase influences the recognition of emotional stimuli (Derntl et al., 2008; Gasbarri et al., 2008; Guapo et al., 2009), state of arousal (Goldstein et al., 2005), and attention (Pilarczyk et al., 2019). Therefore, it is necessary to control for these potential modulating factors, for example by choosing just female participants with regular use of oral contraceptives (OCs). Given that no study to date has taken this approach, it is not clear whether the hypothesis of an SSRI induced modulation of earlier attentional processing is reproducible in a well-powered, healthy female sample with regular OC intake.

The aim of the current study, therefore, is to test whether 7 days of SSRI administration modulates early attentional orienting during emotional processing in healthy female individuals using OCs. In so choosing a healthy sample, we aimed to refine applicability of this hypothesis of SSRI action (Harmer and Cowen, 2013) to an overlooked, yet substantial demographic of SSRI users, with a view toward enhancing applicability to patient samples. To assess the effects of SSRI intake on the interaction between attentional and emotional processing, we administered 20 mg of escitalopram or placebo for 1 week (with an additional assessment after a 1-month wash-out period). We chose 20 mg escitalopram due to its robust blockade of up to 80% of the serotonin transporter (Klein et al., 2006), relatively fast onset of action (Sanchez et al., 2014), higher clinical efficacy (Garnock-Jones and McCormack, 2010), and higher tolerability relative to other common SSRIs (Cipriani et al., 2009). Participants performed a modified version of the emotional flanker task (mEFT) at four time points: before drug administration, after a single dose, after 7 days of SSRI intake, and after a 1-month wash-out period. Unlike previously used paradigms (Harmer et al., 2003; Drueke et al., 2009), the mEFT simultaneously combines attentional and emotional information (Lichtenstein-Vidne et al., 2012). This combination of 1-week of escitalopram intake and our tailored task conditions, therefore, was specifically designed to investigate *early* effects of SSRI intake on the attentional-emotional interplay in an underrepresented sample, namely, female participants with regular OC use. We hypothesized that 1-week escitalopram intake would reduce focus on negative distractor stimuli during task performance, resulting in enhanced selective attention as indicated by disengaging from negative distractors and reduced RT in case of negative distractors, compared to placebo.

## Materials and methods

### Participants

**FIGURE 1:**
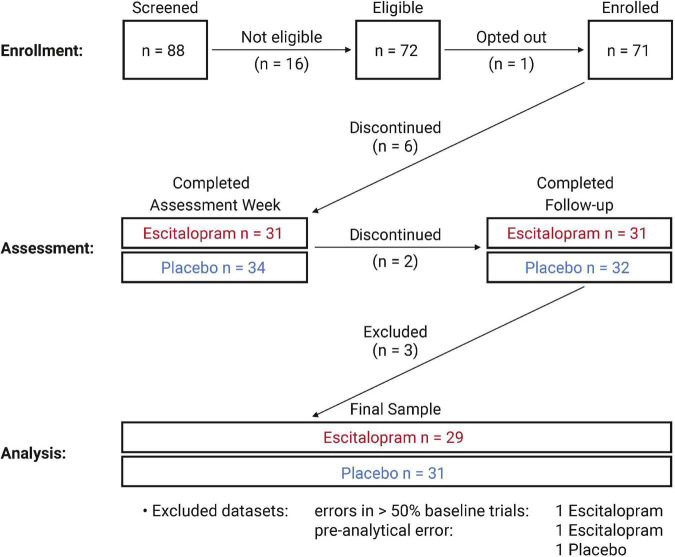
Inclusions and exclusions: The figure depicts the number of participants included in each step of enrollment, assessment, and analysis. Due to self-reported side effects, 6 participants voluntarily discontinued participation during the assessment week (placebo = 2, escitalopram = 4). Two participants did not return for the follow-up assessment. Three participants (placebo = 1, escitalopram = 2) were excluded following implementation of quality control measures.

Participants were recruited from the general public and the local database of the Max Planck Institute for Human Cognitive and Brain Sciences. Eighty-eight participants were rigorously screened, and exclusions were made based on tobacco use, other medication use, presence or history of neurological or psychological disorders, body-mass index (BMI) outside the range of 18.5–25 kg/m^2^, alcohol abuse, or drug abuse. We screened for psychiatric and neurological health using the Structured Clinical Interview for DSM-IV Axis I Disorders (non-patient version) (SCID-I) (First, 2002), the Hamilton Depression Rating Scale (Hamilton, 1960), the Revised NEO Personality Inventory (McCrae et al., 2005) and the Mood Spectrum Self−Report Measure (Dell’Osso et al., 2002). Electrocardiogram (ECG) recordings were used to screen for abnormal QT times. All participants were female, taking estrogen- and progesterone-combined OCs to downregulate pulse frequency of gonadotropin releasing hormone, for at least 3 months prior to participation. By suppressing levels of follicle stimulating hormone (FSH) and luteinizing hormone (LH), OC-use thus prevents follicular development and ovulation (Bastianelli et al., 2018) thereby controlling for sex hormone fluctuations during natural menstrual cycle (Mihm et al., 2011) that may modulate SSRI responsivity (LeGates et al., 2019). In addition, we strictly limited assessments to the pill-interval to control for potential hormonal effects during the pill-free interval. Participants were between 18 and 35 years of age. Seventy-one participants were enrolled, of whom 65 completed the assessment week. Six participants voluntarily discontinued participation during the assessment week. Additionally, two (placebo *n* = 2) participants did not complete a follow-up assessment. Of the 63 participants who completed the study, three were excluded due to QC concerns (Figure 1). All participants were monitored by a physician during the study, provided written informed consent prior to enrollment, and received financial compensation. We obtained ethical approval from the Ethics Committee of the Faculty of Medicine at Leipzig University (approval number 390/16-ek) and conducted all study procedures in accordance with the Declaration of Helsinki of 2013.

### Study design

We administered 20 mg of escitalopram (*n* = 29) or placebo (*n* = 31) to healthy female participants for 7 days. During the administration week, we assessed behavioral responses to an emotional distractor task. Task performance was initially assessed at baseline, prior to escitalopram or placebo intake. Following the baseline measurement, participants were randomly assigned to receive either escitalopram or placebo using a 1:1 allocation method. Both the participant and the experimenter were blind to condition allocation. Behavioral performances were subsequently assessed after a single dose (Day 1), and again following the third assessment, which took place after 7 days (Day 7). Escitalopram and placebo intake occurred at fixed times each day. All participants returned for a follow-up assessment following a 4–6-week follow-up, in which no escitalopram or placebo was administered. ECG recordings were conducted at Days 1, 4, 7, and follow-up, to monitor QT intervals. Adverse reactions to escitalopram were recorded using the antidepressant side-effects checklist (ASEC) (Uher et al., 2009). Changes in mood and anxiety were recorded with different psychological inventories: the POMS (German language version of the profile of mood spectrum) (McNair et al., 1981), a validated (Gibson, 1997) self-report measure assessing various mood status, the DAS (Dysfunctional attitude scale) (Weissman and Beck, 1978), a validated (Weissman, 1979; de Graaf et al., 2009) questionnaire to assess negative attitudes and cognitive vulnerability and the STAI (State trait anxiety index) (Spielberger et al., 1983) validated (Guillén-Riquelme and Buela-Casal, 2014) measuring both state and trait anxiety, transient reactions in specific situations, and specific attributes of personality.

### Task procedure and primary outcome measure

**FIGURE 2:**
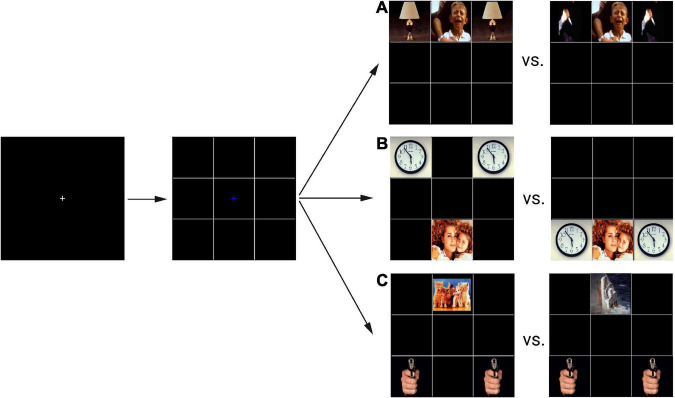
Visual stimulus of the emotional distractor task. Depicted here is an example of a typical trial. Participants view a fixation cross which is then replaced by a second fixation cross in a 9-panel grid. Both the target picture and the distractors subsequently appear, prompting participants to indicate the target picture location, while ignoring distracting flanker pictures. The different conditions of this panel are shown: flankers were neutral or negative **(A)** and incongruent or congruent to the target pictures **(B)**; targets were either positively or negatively valenced **(C)**. Target location was either above or below the previously shown fixation cross. Ms, milliseconds. Images are from The International Affective Picture System (IAPS).

We assessed emotional and attentional responses using a mEFT (Lichtenstein-Vidne et al., 2012), implemented in EPrime 2.0 professional (Stahl, 2006) running on the Windows XP operating system. Visual task stimuli were obtained from the International Affective Picture System (IAPS; Bradley et al., 2001). The experiment contained 3 blocks of 32 trials each, resulting in 96 trials. Participants were instructed to indicate whether a target picture appeared above or below a fixation cross, via a keypress, while ignoring flanker images. In each trial, peripheral distracting pictures contained negative or neutral emotional valences while targets were either negative or positive. Participants were asked to respond as quickly and accurately as possible. The outcome measure was reaction time (RT), measured in milliseconds (ms), and calculated as the difference between the time of target onset and participant response. Three independent within-subject variables were manipulated: flanker location congruency with the target picture (congruent, incongruent), flanker valence (negative, neutral), and target valence (negative, positive) (Figure 2).

### Data analysis

#### Demographics and mood assessment

Statistical analyses were conducted with the Statistical Package for the Social Sciences (SPSS, v24). Peripheral plasma escitalopram levels were quantified by high-performance chromatography using quality control (QC) sample (Teichert et al., 2020). Moreover, we measured plasma levels of FSH and LH to assess regular OC-induced suppression of these hormones. We used independent sample *t*-tests to assess potential group differences in age, BMI, and downregulated hormonal profiles. ASEC scores at both single dose and steady state were also assessed with independent sample *t*-tests. Potential changes in mood and anxiety, as recorded by the DAS, STAI, and POMS were each assessed separately using a 2 × 3 analysis of variance (ANOVA) with *group* and *time* as factors. Results of mood and anxiety analyses were considered statistically significant at a Bonferroni-corrected α-level of *p* < 0.016 to account for multiple testing (0.05/3).

#### Data preprocessing and quality control

We preprocessed all data using EPrime (version 2.0). Each assessment contained 10 practice trials that were excluded during pre-processing. To identify outlier trials, we calculated the mean and standard deviation for each trial. Individual outlier trials were removed with SPSS, using a cutoff of ± 2.5 standard deviations from the mean (2% of all trials for all participants). Additionally, only trials in which a correct response was given were included (98% of all trials of all participants).

#### Validation of task performance

Prior to analyses comparing SSRI and placebo conditions, we performed a control analysis to confirm that participants understood task instructions. To this end, we assessed performance at baseline in all participants who completed this measurement (*n* = 71) and passed data QC, without reference to group allocation or to further exclusion. In line with previous studies with this task (Lichtenstein-Vidne et al., 2012), we specified an ANOVA to test for a main effect of congruency, a main effect of flanker valence, and for an interaction between congruency and target valence.

#### Analysis of attentional and valence-dependent task performance

To test our main hypothesis, we employed a five-way repeated measures mixed model analysis of variance (ANOVA) in SPSS. Here, we specified 5 independent variables; *group* (escitalopram, placebo), *time* (baseline, Day 1, Day 7, Follow-up), *target valence* (positive, negative), *flanker valence* (negative, neutral), and *congruency* (congruent, incongruent). We specified *group* as the between subjects factor, *time* as the within subjects factor and RT in each of the target, flanker valence, and congruency conditions as the dependent variable.

#### Correlation between peripheral plasma escitalopram levels and modified version of the emotional flanker task performance

We tested potential correlations between peripheral measures of plasma escitalopram and behavioral performance using a bivariate Pearson’s correlation implemented in SPSS. Here we correlated peripheral plasma levels acquired at day 7 of escitalopram intake with the mean performance in task performance during each of the valence and congruency conditions in the escitalopram group only (*n* = 29). We employed 6 models to assess a potential association between (i) congruency (congruent and incongruent RT), (ii) flanker valence (Negative and Neutral RT) and (iii) target valence conditions (Negative and Positive RT). Results were considered statistically significant at a Bonferroni corrected threshold of *p* < 0.008 (0.05/6) to account for multiple testing.

#### Bayesian analysis

Finally, we employed a Bayesian estimation to assess the likelihood of the null hypothesis in each task condition. To this end, we used JASP (v0.12.2—JASP Team, 2020) to implement a Bayesian repeated measures ANOVA for each condition in all cognitive and valence specific analyses [i.e., one Bayesian approach for each of the congruency (*congruent/incongruent)*, target valence (*negative/positive*), and flanker valence (*negative/neutral*) conditions], resulting in 6 Bayesian estimations in total.

## Results

### Demographics

Analyses of demographic variables yielded no significant group differences on any baseline control measures. Group comparisons of time between final escitalopram or placebo intake and follow-up measurement also indicated no significant differences. Escitalopram levels were consistent with previously reported data (Rao, 2007). Analyses of ASEC scores indicated a significant group difference in mean self-reported side effects at single dose (*t* = −3.389, *p* = 0.001) but not at steady state (*t* = −0.675, *p* = 0.502). Ovulation inhibition was confirmed as suppressed via gonadotropins measurement (Goldzieher et al., 1970; Table 1). Estrogen- and progesterone-combined hormonal contraceptives taken by the participants are listed in Table 2.

**TABLE 1:** Demographic analysis overview: Baseline and demographic variables across both groups.

Demographics	Escitalopram (*M* ± *SD*)	Placebo (*M* ± *SD*)	*t*-value	*p*-value
Age (years)	24 ± 3	23 ± 4	0.99	0.33
BMI (kg/m^2^)	22 ± 1.7	21 ± 1.7	1.08	0.28
Lutropin (μ/l)	2.0 ± 2.7	1.4 ± 2.0	0.92	0.34
Follitropin (μ/l)	2.9 ± 3.2	2.1 ± 3.0	0.99	0.33
Escitalopram single dose (ng/ml)	20 ± 5	n.d.	−	−
Escitalopram steady state (ng/ml)	46 ± 11	n.d.	−	−
Time to follow-up (days)	33 ± 5	35 ± 7	1.37	0.18

Results show no difference on any baseline or demographic measure, nor on the time in between the completion of the assessment week and the onset of the follow-up measurement. Values refer to mean and standard deviation.

kg/m^2^, kilogram force per square meter; u/l, units per liter; ng/ml, nanograms/milliliters.

**TABLE 2:** Contraceptive usage.

Group	Number of participants	Compound (dose)
Placebo	12	Ethinylestradiol 0.03 mg (dienogest 2 mg)
	4	Ethinylestradiol 0.03 mg (chlormadinonacetat 2 mg)
	1	Ethinylestradiol 0.03 mg (desogestrel 0.15 mg)
	4	Ethinylestradiol 0.02 mg (levonorgestrel 0.1 mg)
	5	Ethinylestradiol 0.03 mg (levonorgestrel 0.15 mg)
	1	Ethinylestradiol 0.02 mg (desogestrel 0.15 mg)
	3	Ethinylestradiol 0.03 mg (levonorgestrel 0.125 mg)
	1	Ethinylestradiol 2.7 mg (etonogestrel 11.7 mg)
Escitalopram	2	Ethinylestradiol 0.02 mg (levonorgestrel 0.1 mg)
	2	Ethinylestradiol 0.03 mg (levonorgestrel 0.15 mg)
	1	Ethinylestradiol 0.02 mg (desogestrel 0.15 mg)
	3	Ethinylestradiol 0.03 mg (chlormadinonacetat 2 mg)
	17	Ethinylestradiol 0.03 mg (dienogest 2 mg)
	1	Ethinylestradiol 0.02 mg (levonorgestrel 0.1 mg)
	1	Ethinylestradiol 0.03 mg (levonorgestrel 0.125 mg)
	1	Ethinylestradiol 0.03 mg (desogestrel 0.15 mg)
	1	Ethinylestradiol 0.02 mg (drospirenon 3 mg)

Listed is an overview of taken hormonal contraceptives from the study participants. All participants used combined contraceptives to inhibit ovulation.

### Mood and anxiety monitoring

We did not observe group differences on measures of either (i) state anxiety [time: *F*(1, 55) = 1.939, *p* = 0.154, partial eta^2^ = 0.066; time by group: *F*(1, 55) = 2.941; *p* = 0.061; partial eta^2^ = 0.097; group: *F*(1, 1) = 0.665, *p* = 0.418, partial eta^2^ = 0.012], or (ii) mood [DAS: time: *F*(1, 59) = 2.374, *p* = 0.102, partial eta^2^ = 0.074; time by group: *F*(1, 59) = 1.024, *p* = 0.365, partial eta^2^ = 0.034; group: *F*(1, 1) = 0.812, *p* = 0.371; partial eta^2^ = 0.013]; POMs [time: *F*(1, 60) = 7.207, *p* = 0.002, partial eta^2^ = 0.194; time by group: *F*(1, 60) = 1.145, *p* = 0.325, partial eta^2^ = 0.037; group: *F*(1, 1) = 0.396, *p* = 0.531, partial eta^2^ = 0.006].

### Baseline replication analysis

Analysis of baseline (*n* = 71) performance yielded an outcome comparable to previous studies, with a significant main effect for location congruency [*F*(1, 70) = 42.306; *p* < 0.001; partial eta^2^ = 0.377], represented by faster RTs in congruent vs. incongruent trials. Additionally, results showed a congruency by flanker valence interaction [*F*(1, 70) = 4.570; *p* = 0.036; partial eta^2^ = 0.061], with slower RTs in congruent trials during negative flankers as compared to the neutral condition. In contrast to previous findings, however, we did not observe a significant effect of distractor valence [*F*(1, 70) = 1.758; *p* = 0.189; partial eta^2^ = 0.024] nor target valence [*F*(1, 70) = 2.942; *p* = 0.091; partial eta^2^ = 0.040].

### Task performance analysis over time

#### Five-way analyses of group performance over time

**FIGURE 3:**
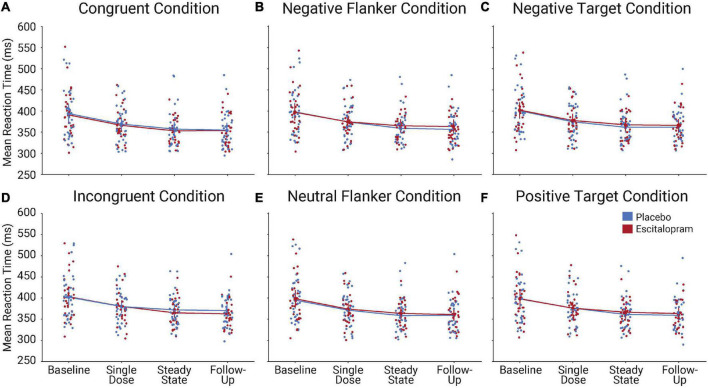
Timeline of behavioral performance during 1-week of escitalopram-intake and after a 1-month wash-out period for each task condition: We measured RT [in milliseconds (ms)] performance on the mEFT in both the escitalopram and placebo groups at baseline, single dose, after 1-week of continuous intake, and following a 1-month wash-out period. We assessed performance in each of the **(A)** congruent and **(B)** incongruent conditions, **(C)** the negative and **(D)** neutral flanker conditions, and **(E)** the negative target and **(F)** positive target conditions. While results indicate a significant effect of time, with decreasing RT between the baseline and follow-up assessments, we did not observe any indication of a difference between groups on any measure of performance.

Our results show a significant effect of time [*F*(1, 3) = 33.163; *p* < 0.001; partial eta^2^ = 0.364], with decreased RTs during performance of each target valence, flanker valence, and congruency condition over the course of the experiment. However, there was no significant group by time effect [*F*(1, 3) = 0.128; *p* = 0.943; partial eta^2^ = 0.002]. Analysis of a congruency effect show significance [*F*(1, 1) = 127.753; *p* < 0.001; partial eta^2^ = 0.688], but we did not observe a significant group by congruency interaction [*F*(1, 1) = 0.071; *p* = 0.791; partial eta^2^ = 0.001]. Complimentary analysis of the flanker valence conditions (*negative, neutral*) shows no significant effect [*F*(1, 1) = 1.176; *p* = 0.283; partial eta^2^ = 0.020] nor an interaction effect with group [*F*(1, 1) = 0.007; *p* = 0.932; partial eta^2^ = 0.000]. Analysis of target valence conditions (*negative, positive*) yields no significant effect [*F*(1, 1) = 3.582; *p* = 0.063; partial eta^2^ = 0.058] and no group interaction [*F*(1, 1) = 0.386; *p* = 0.537; partial eta^2^ = 0.007] (Figure 3).

#### Correlation analyses

**FIGURE 4:**
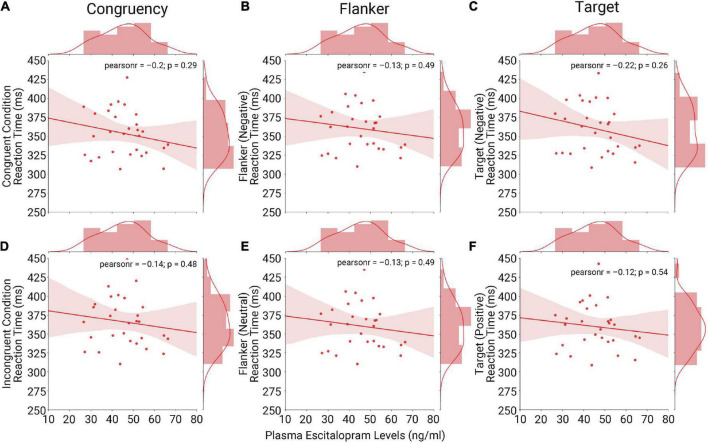
Correlation analyses at day seven of escitalopram intake for each task condition: We assessed a potential correlation between peripheral measures of plasma escitalopram and behavioral performance in each task condition category with a bivariate Pearson’s correlation. Results for the congruency condition [left column **(A,D)**], flanker condition [middle column **(B,E)**] and target condition [right column **(C,F)**] show no evidence of a correlation. Results considered significant at a Bonferroni-corrected *p*-value of < 0.008 due to multiple testing.

Results of a bivariate correlation analyses within the escitalopram group only do not suggest any apparent relationship between peripheral plasma escitalopram levels, acquired at the final day of intake, with behavioral performance in each of the specific task conditions at the seventh day of intake. Across six correlation analyses for each condition within the congruency, flanker valence, and target valence conditions, peripheral plasma escitalopram did not correlate with performance (all *p* > 0.008) (Figure 4).

### Bayesian analyses

A series of Bayesian repeated measures ANOVAs show moderate to strong evidence in favor of the null hypothesis for each cognitive and emotional behavioral outcome, compared to the alternative hypothesis (Table 3). Model comparisons show increasing likelihood in favor of the null hypothesis for each additional model contribution (i.e., of the group and group by time interaction terms). Consistent with frequentist repeated measures analyses, no evidence for the null hypothesis is observed for the *time* factor.

**TABLE 3:** Results of Bayesian analysis for each cognitive and valence domain: Repeated measures Bayesian analyses show moderate to strong evidence for the null hypothesis when considering the group and group by time interaction terms for each cognitive and valence-dependent condition.

*Congruency*	P (M)	BF (M)	BF_01_	Error (%)	*Incongruency*	P (M)	BF (M)	BF_01_	Error (%)
Time	0.2	9.42	1	−	Time	0.2	9.57	1	−
Time + group	0.2	1.60	2.4	3.52	Time + group	0.2	1.50	2.58	3.44
Time × group	0.2	0.04	63.5	6.18	Time × group	0.2	0.08	33.09	6.30
*Flanker negative*					*Flanker neutral*				
Time	0.2	9.45	1	−	Time	0.2	10.20	1	−
Time + group	0.2	1.58	2.47	3.41	Time + group	0.2	1.43	2.72	3.52
Time × group	0.2	0.05	54.52	6.56	Time × group	0.2	0.07	40.54	6.39
*Target negative*					*Target positive*				
Time	0.2	10.79	1	−	Time	0.2	9.52	1	−
Time + group	0.2	1.38	2.83	3.49	Time + group	0.2	1.54	2.52	3.53
Time × group	0.2	0.05	56.81	6.20	Time × group	0.2	0.06	42.13	6.65

Results indicate the likelihood of the null hypothesis as approximately twice that of the alternative hypothesis for the group factor, and several times that of the alternative hypothesis for the interaction term. P (M), prior model plausibility; BF (M), posterior model odds; BF_01_, Bayes factor likelihood of the null hypothesis compared to the alternative hypothesis; Error (%), Error computation of Bayes factor.

## Discussion

In the present study, we assessed whether 1-week administration of escitalopram modulates the attention-emotion interaction in healthy female participants with regular OC use. Using a mEFT, we tested the hypothesis that escitalopram intake would facilitate the disengagement from negatively valenced task distractors (Harmer et al., 2009a). Against our hypothesis, our results do not suggest an effect of escitalopram on emotional and attentional distraction, either after single dose, or after 7 days continuous intake, or at a 1-month follow-up assessment. While we observed a significant improvement in task performance over time with a decrease in RT to task stimuli, there was no observed significant difference in performance between groups. Moreover, Bayesian analyses yield moderate to strong support in favor of the null, relative to the alternative, hypothesis. Consequently, these results do not suggest an effect of 1-week escitalopram intake on selective attention and inhibition of negatively valenced distractors in this sample of healthy female participants on OCs.

One possible reason for our findings may be the specificity of our task demands. Specifically, unlike previous emotional processing tasks, the mEFT combines emotional and attentional domains using peripheral distractors that simultaneously present emotional and spatial information (Lichtenstein-Vidne et al., 2012). Previous studies, however, typically employed tasks that considered these domains individually, such as a facial recognition task (Harmer et al., 2003) or an attentional network task (Drueke et al., 2009). Furthermore, previous findings were mainly obtained using tasks that present distracting emotional information inside the focus of attention, such as the emotional Stroop task and the dot-probe task. For example, Browning et al. (2007a), employed a visual probe task to show an increase in attention to socially relevant positive words after a single dose of citalopram. In another study, Murphy et al. (2009b) showed reduced attentional orienting to threatening stimuli after 1-week citalopram intake with the use of an attentional probe task. Unlike these paradigms, however, our task presented the distracting emotional information *outside* the focus of attention. Therefore, while previous studies provide evidence for a modulation of an attentional bias toward distracting emotional content that is presented inside the focus of attention, we investigated the effect of escitalopram on an attentional bias to task-irrelevant emotional content, when task settings do not encourage distractors’ processing. Investigating such an effect is also of special interest, considering that only a small portion of the visual stimuli in everyday life appear at the visual center (Wandell, 1995). Consequently, our results do not indicate an effect of escitalopram on selective attentional processing of task irrelevant emotional and task relevant spatial information in healthy females on OCs. Our findings are consistent with the concept that emotional processing is not automatic, but highly task and stimuli dependent. Future studies comparing the difference of task-relevant and irrelevant distractors in emotional processing tasks are required, however, to directly assess this possibility.

Another possible reason may be a product of the emotional stimuli presented as part of the mEFT. Evidence suggests that emotional recognition tasks that employ facial stimuli, as used by Ahmed et al. (2021), may be particularly sensitive to changes in emotional processing compared to that of the mEFT, which primarily presents situational stimuli. This interpretation is consistent with other findings that did not suggest an effect of linguistic stimuli (Browning et al., 2019; Ahmed et al., 2021) on emotional processing, stimuli which are thought to have a lower emotional impact relative to pictorial stimuli (Lees et al., 2005). While such illustrations have been described as being less emotionally salient compared to the real-life pictures, presented in our task (Okon-Singer et al., 2013), it is likely that facial stimuli may uniquely stimulate specific brain regions (Britton et al., 2006). In sum, our findings, while not replicating previous findings in healthy volunteers, are consistent with the hypothesis that certain emotional stimuli maybe somewhat specific to certain task demands. Larger studies with a wider array of emotionally salient stimuli are needed to further explore this possibility, however.

Given our longitudinal design and the fact that participants performed the task multiple times, our results may also show a practice or habituation effect. Though we observed no difference in performance between groups, we did observe a significant effect of time with decreased RT, suggesting that all participants responded faster over the course of the experiment. Participants were asked to respond via a simple button press, making the task relatively easy to perform. With a healthy sample showing no aberrant emotional processing (as measured by our baseline replication analysis), it is possible that participants simply adapted or habituated to the presented stimuli. One possible counterpoint, however, is that the presented stimuli where randomized across each measurement, suggesting that it is likely that different stimuli were presented at each assessment day. However, as this randomization of stimuli was random, we cannot rule out the possibility that similar stimuli were shown or repeated at different times during the experiment. As a result, the simpler nature of our task in the presence of a healthy sample may have dampened an already small effect of the SSRI, thus leading to our observed outcomes. However, it is also worth mentioning that our task procedure was specifically designed in this less complex manner, given that existing evidence has shown that increased task difficulty may conflict with emotional processing (Pessoa, 2005; Cohen et al., 2011). Regardless, a simple task contributing to habituation and practice effects in a healthy sample present a viable interpretation for our null result, an interpretation which future studies in patients and with more demanding tasks may address.

One final, though admittedly more speculative possibility, is the influence of downregulated endogenous sex hormones via OC use and the associated effects on SSRI responsivity. As we confirmed OC- induced suppression of LH and FSH in comparison to natural cycling women and to control for sex hormone fluctuations during the menstrual cycle (Mihm et al., 2011), it is arguable that this induced downregulation of endogenous sex hormones (Givens et al., 1976) may have dampened, at least in part, the effects of escitalopram on our primary outcome measure, the behavioral response to the mEFT. Evidence in favor of this possibility comes from previous studies showing endogenous estradiol modulates serotonergic transmission, SSRI responsivity and affect (Amin et al., 2005; Michopoulos et al., 2011; Ocampo Rebollar et al., 2017). However, we stress that, as we did not investigate estradiol or an interaction effect of SSRIs, OCs and time on task performance, this interpretation should be taken with great caution. Future interventional studies with multiple groups are required to explore this possibility further.

There are also several limitations to this study that should be considered. First, our results are limited to healthy participants only as we did not include any patients in our sample. This decision was deliberate, however, as we aimed to replicate previous findings in healthy participants and to extend these findings to an underrepresented demographic. While future studies in clinical populations are crucial, our analyses in healthy female participants with long term OC-use provides a much needed extension of previous preclinical studies. Secondly, we cannot exclude that a longer drug administration duration would have resulted in differential effects given that, in patients, antidepressants often take up to 3–4 weeks to exhibit clinically relevant changes in mood (Frazer and Benmansour, 2002). Nevertheless, we again explicitly chose this time frame of administration in order to test our hypothesis of *earlier* alterations in attentional processing in response to SSRIs, which is hypothesized to *precede* the changes in mood often seen at later stages of administration (Harmer and Cowen, 2013). Thirdly, the interpretation of our findings may be limited to escitalopram and thus, we cannot make any comments regarding the potentially differential effects of other SSRIs as escitalopram, unlike other common SSRIs such as paroxetine or fluoxetine, exhibits a unique allosteric binding affinity for the serotonin transporter (Klein et al., 2006). As a result, future studies are needed to assess whether our findings extend to a class-effect or are specific to escitalopram at this specific dose. Fourth, we acknowledge that more experimental groups, in which naturally cycling females are investigated with their endogenous sex hormone fluctuations, would be necessary to clarify whether OC-use, specifically, contributed to our findings. As such, we can only speculate, with great caution, that this is a contributing factor. Future studies with different SSRIs, varying doses, hormonal measures and administration regimes (Frye, 2006), and multiple groups that are specifically designed to assess this possibility, are necessary in order to further discuss this possibility. Finally, our analyses are limited to behavioral outcomes only, which differs to previous studies that assessed neural responses during combined SSRI intake and task performance (Harmer et al., 2006; Murphy et al., 2009a; Norbury et al., 2009). In contrast, we did not assess neural responses to escitalopram during mEFT performance. Future studies employing similar samples (and patient populations) should also consider functional neuroimaging to investigate regional and global effects of escitalopram intake at the neural level during attentional and emotional processing.

In conclusion, our results do not indicate an effect of 7 days escitalopram intake on attentional-emotional interaction in healthy female participants taking OCs. While these outcomes may be a result of the specific task requirements of the modified emotional flanker task, or of our chosen sample of healthy volunteers, another possible explanation may be practice effects resulting from simple task requirements. Nevertheless, our findings provide much needed data on a highly relevant, yet underrepresented sample, thus making a critical contribution toward refining the attention-emotion cognitive model of antidepressant action in healthy volunteers. As a result, these results provide a solid platform for studies in patients, with the ultimate goal of improving personalized treatment for depression.

## Data availability statement

The raw data supporting the conclusions of this article will be made available by the authors, without undue reservation.

## Ethics statement

The studies involving human participants were reviewed and approved by the Ethics committee of the medical faculty of the University of Leipzig. The patients/participants provided their written informed consent to participate in this study.

## Author contributions

NB, EM, RZ, and TR contributed to data analysis. NB, EM, RZ, TR, JS, and HO-S contributed on manuscript writing process. NB, EM, RZ, JS, HO-S, FP, US, TR, GZ, RR, and AV contributed on reviewing the manuscript. All authors contributed to the article and approved the submitted version.
